# Transgenic apple plants overexpressing the *chalcone 3*-*hydroxylase* gene of *Cosmos sulphureus* show increased levels of 3-hydroxyphloridzin and reduced susceptibility to apple scab and fire blight

**DOI:** 10.1007/s00425-016-2475-9

**Published:** 2016-02-19

**Authors:** Olly Sanny Hutabarat, Henryk Flachowsky, Ionela Regos, Silvija Miosic, Christine Kaufmann, Shadab Faramarzi, Mohammed Zobayer Alam, Christian Gosch, Andreas Peil, Klaus Richter, Magda-Viola Hanke, Dieter Treutter, Karl Stich, Heidi Halbwirth

**Affiliations:** Institute of Chemical Engineering, Technical University of Vienna, Getreidemarkt 9, 1060 Vienna, Austria; Federal Research Centre for Cultivated Plants, Institute of Breeding Research on Horticultural and Fruit Crops, Julius Kühn-Institut, Pillnitzer Platz 3a, 01326 Dresden, Germany; Unit of Fruit Science, Technical University of Munich, Dürnast 2, 85350 Freising, Germany; Chair of Urban Water Systems Engineering, Technical University of Munich, Am Coulombwall, 85748 Garching, Germany; Federal Research Centre for Cultivated Plants, Institute for Resistance Research and Stress Tolerance, Julius Kühn-Institut, Erwin-Baur-Str. 27, 06484 Quedlinburg, Germany

**Keywords:** Chalcone 3-hydroxylase (CH3H), Dihydrochalcones, *Erwinia amylovora*, 3-Hydroxyphloretin, 3-Hydroxyphloridzin, *Malus* × *domestica*, *Venturia inaequalis*

## Abstract

**Overexpression of chalcone-3-hydroxylase provokes increased accumulation of 3-hydroxyphloridzin in*Malus*. Decreased flavonoid concentrations but unchanged flavonoid class composition were observed. The increased 3-hydroxyphlorizin contents correlate well with reduced susceptibility to fire blight and scab.**

The involvement of dihydrochalcones in the apple defence mechanism against pathogens is discussed but unknown biosynthetic steps in their formation hamper studies on their physiological relevance. The formation of 3-hydroxyphloretin is one of the gaps in the pathway. Polyphenol oxidases and cytochrome P450 dependent enzymes could be involved. Hydroxylation of phloretin in position 3 has high similarity to the B-ring hydroxylation of flavonoids catalysed by the well-known flavonoid 3′-hydroxylase (F3′H). Using recombinant F3′H and chalcone 3-hydroxylase (CH3H) from *Cosmos sulphureus* we show that F3′H and CH3H accept phloretin to some extent but higher conversion rates are obtained with CH3H. To test whether CH3H catalyzes the hydroxylation of dihydrochalcones *in planta* and if this could be of physiological relevance, we created transgenic apple trees harbouring *CH3H* from *C. sulphureus*. The three transgenic lines obtained showed lower polyphenol concentrations but no shift between the main polyphenol classes dihydrochalcones, flavonols, hydroxycinnamic acids and flavan 3-ols. Increase of 3-hydroxyphloridzin within the dihydrochalcones and of epicatechin/catechin within soluble flavan 3-ols were observed. Decreased activity of dihydroflavonol 4-reductase and chalcone synthase/chalcone isomerase could partially explain the lower polyphenol concentrations. In comparison to the parent line, the transgenic *CH3H*-lines showed a lower disease susceptibility to fire blight and apple scab that correlated with the increased 3-hydroxyphlorizin contents.

**Electronic supplementary material:**

The online version of this article (doi:10.1007/s00425-016-2475-9) contains supplementary material, which is available to authorized users.

## Introduction

**Fig. 1:**
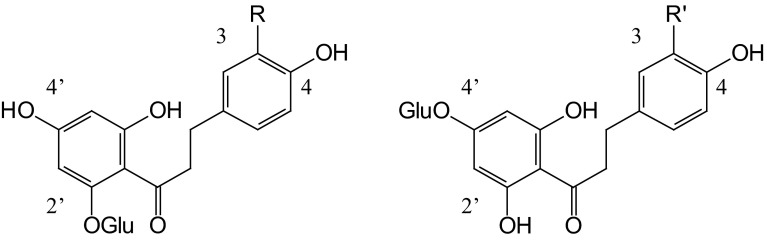
Main phloretin derivatives found in *Malus* species. R=H: phloretin. R=OH: 3-hydroxyphloretin. R′=H: trilobatin. R′=OH: sieboldin

Apple (*Malus* × *domestica* Borkh.) belongs to the most popular fruits world-wide and their consumption is suggested to be health-beneficial (Boyer and Liu 2004; Ehrenkranz et al. 2005; Hyson 2011). Apple is unique among plant species because it produces large amounts of dihydrochalcones, predominantly phloridzin (phloretin 2′-*O*-glucoside) (Fig. 1), which are not or only found in low levels in other plants. Some *Malus* species accumulate the 4′-*O*-glucosides trilobatin (*M. trilobata* C. K. Schneid.) and sieboldin (*M. sieboldii* Rehder) besides the 2′-*O*-glucosides (Fig. 1) (Williams 1982; Gaucher et al. 2013b). The physiological function of dihydrochalcones in apple remains, however, unclear. A presumed involvement in pathogen defence has been divergently discussed, but no final proof was presented so far (Gosch et al. 2010a, b). Although dihydrochalcones have antimicrobial activity (MacDonald and Bishop 1952; Barreca et al. 2014), their constitutive presence in apple tissues alone does not seem to be decisive for disease resistance (Picinelli et al. 1995). Hydroxylation in position 3 is the first step in the oxidation of phloretin by polyphenol oxidases (PPO), which results in the formation of highly reactive quinoid structures that can interfere with cell invading pathogens (Le Guernevé et al. 2004; Guyot et al. 2007; Overeem 1976). The relevance of PPO and peroxidase reaction products such as 3-hydroxyphloretin, dihydrochalcone dimers and quinoid structures and of their formation speed was therefore discussed as well (de Bernonville et al. 2010, 2011; Gaucher et al. 2013a, b). In the case of the fire blight resistant crabapple cv. Evereste, however, a correlation between pathogen resistance and the constitutive presence of sieboldin was not observed (de Bernonville et al. 2011). Whereas dihydrochalcone biosynthesis has been studied in the past few years (Jugdé et al. 2008; Gosch et al. 2009, 2010a; Dare et al. 2013; Ibdah et al. 2014), the introduction of a hydroxyl group in position 3 of dihydrochalcones was not elucidated so far. Two types of enzymes could catalyse the reaction, PPOs and/or cytochrome P450 dependent monooxygenases. Although PPOs from apple have repeatedly been reported to produce 3-hydroxyphloretin as intermediates in the phloretin oxidation (Goodenough et al. 1983; Haruta et al. 1998; Ridgway and Tucker 1999), their physiological relevance for the 3-hydroxyphloridzin biosynthesis in apple leaves has not yet been demonstrated. It seems however unlikely that such an unspecific enzyme which produces a spectrum of cell-toxic compounds should be involved in the biosynthesis of the 3-hydroxyphloridzin that is constitutively present in *Malus* sp.

**Fig. 2:**
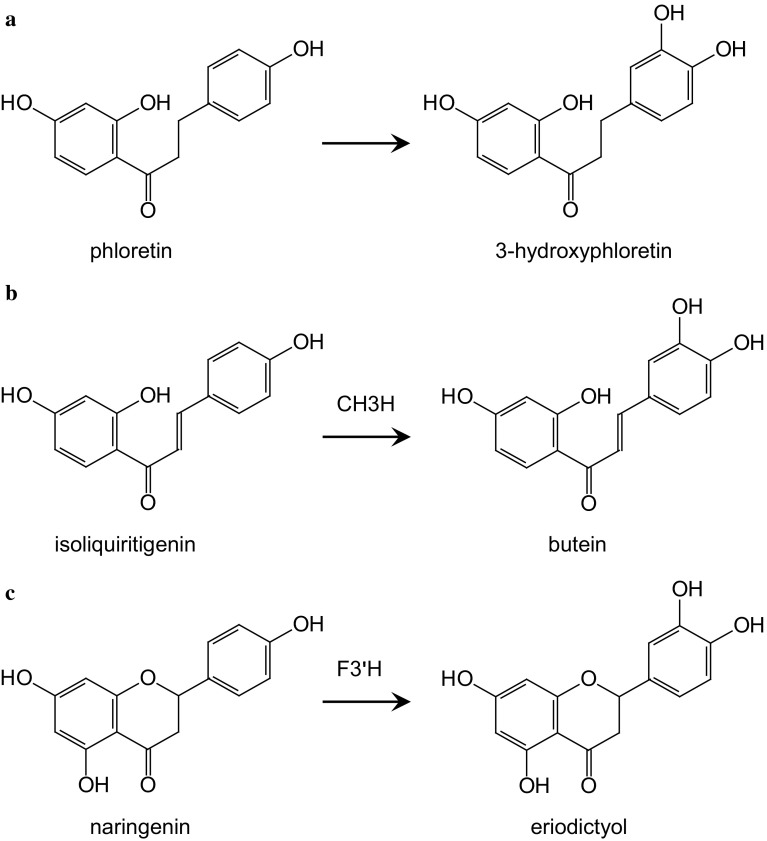
Hydroxylation of dihydrochalcones (**a**) and chalcones (**b**) in position 3 and flavonoids in position 3′ (**c**)

Hydroxylation in position 3 of dihydrochalcones shows high similarity to the introduction of a second hydroxyl group in the B-ring of flavonoids and chalcones (Fig. 2) which are catalysed by the cytochrome P450 dependent monooxygenases flavonoid 3′-hydroxylase (F3′H) and chalcone 3-hydroxylase (CH3H) (Schlangen et al. 2010b). The chemical structure of dihydrochalcones is closely related to that of chalcones as both lack the heterocyclic ring that is typically found in flavonoid structures (ring C) (Fig. 2). Whereas CH3H depends on specific motifs in the protein structure and was so far only described in Asteraceae species, F3′H can be found in many plant species (Schlangen et al. 2009, 2010a). In *Malus* sp., at least 3 genes encoding F3′Hs have been identified (Han et al. 2010) and it cannot be excluded that they are involved in the 3-hydroxyphloridzin biosynthesis besides their essential role in the flavonoid pathway. In the present study we tested whether CH3H of the ornamental plant *C. sulphureus* (CsCH3H) accepts phloretin as substrate and we show that constitutive overexpression of *CsCH3H* in apple leaves results in plants with increased 3-hydroxyphloridzin formation which seems to be correlated to reduced susceptibility to the biotic diseases scab and fire blight which are caused by *Venturia inaequalis* (Cooke) G. Winter and *Erwinia amylovora* (Burrill) Winslow et al., respectively.

## Materials and methods

### Chemicals

[^14^C ]-Phloretin was synthesized as described previously (Halbwirth et al. 2006).

3-Hydroxyphloretin was purchased from Apin Chemicals (http://www.apinchemicals.com). 2,2-Diphenyl-1-picrylhydrazyl (DPPH) and 6-hydroxy-2,5,7,8-tetramethylchroman-2-carboxylic acid (trolox) were obtained from Sigma-Aldrich (www.sigmaaldrich.com/austria.html).

#### Production and in vitro testing of recombinant *CsCH3H*

Heterologous expression of *CsCH3H* and *F3′H* in yeast and testing of substrate acceptance was performed as described previously (Schlangen et al. 2010b).

### Vector construction for plant transformation

The binary plasmid vector p9u10-35S::*CH3H* was provided by DNA Cloning Service e.K. (http://dna-cloning.com) using a cDNA clone of *C. sulphureus* (NCBI accession FJ216429) and the p9u10-35S plasmid that contains the *nptII* selectable marker gene driven by the ubiquitin promotor from *A. thaliana*. The cDNA clone was ligated between the *Spe*I and *Mlu*I restriction sites using the primers SpeCH3H and CH3HMlu (Suppl. Table S1).

### Plant material

Proliferating axillary shoot cultures of PinS (JKI, Dresden-Pillnitz, Germany), a seedling of the apple (*Malus* × *domestica* BORKH.) cv. ‘Pinova’ were used for plant transformation. Transformation was performed using the *Agrobacterium tumefaciens* strain EHA105 (Hood et al. 1993) containing the binary plasmid vector p9u10-35S::*CH3H*. Regeneration, selection of transgenic plants, rooting and acclimatization were carried out as previously described (Li et al. 2007). For the studies on gene expression, enzyme activities and polyphenol contents, the first (L1), second (L2) and third (L3) leaves of the shoots were collected separately, shock-frozen in liquid nitrogen and stored at −80 °C until use. Three independent samples were harvested.

### RT-PCR and qRT-PCR analysis

Young leaves of greenhouse-grown shoots (transgenic and non-transgenic) were collected and frozen in liquid nitrogen. The total RNA from leaf tissue was extracted using the InviTrap Spin Plant RNA Mini Kit (Stratec Biomedical AG, Berlin, Germany). First strand cDNA synthesis was performed as described by Flachowsky et al. (2007). Transcripts of *nptII* and *CH3H* were detected by reverse transcriptase (RT)-PCR using the primers nptII_F/R for *nptII* and SpeCH3H/CH3HMlu for CH3H.

The relative mRNA expression level of genes of the flavonoid genes encoding *ANS*, *ANR* and *FGT* was determined by quantitative Real-Time PCR SYBR^®^ Green PCR Master Mix according to the supplier’s instruction on a StepOnePlus system (Applied Biosystems, Darmstadt, Germany) with first-strand cDNA as template using the primers listed in Suppl. Table S1.

### Southern blots

Southern hybridization experiments were performed using 10 µg of DNA digested with 100 U *Sal*I (Life Technologies, Darmstadt, Germany) at 37 °C overnight. The restricted DNA was separated on a 0.8 % agarose gel in 1× Trisacetate-EDTA (TAE) buffer and blotted onto a positively charged nylon membrane (Roche Deutschland Holding GmbH, Mannheim, Germany) by capillary transfer (Southern 1975) with 20× SSC (0.15 M NaCl, 0.015 M tri-sodium citrate dihydrate, pH 7.0) as transfer buffer. The membrane was hybridized with PCR-amplified, digoxygenin-labeled probes of *nptII* and *CH3H*, respectively. Probes were amplified using the PCR DIG Probe Synthesis Kit (Roche). Amplification was performed using the primers nptII_F/R for *nptII* and SpeCH3H/CH3HMlu (Suppl. Table S1) for *CH3H*, respectively. Detection was performed using Anti-DIG-AP (Roche) and ECF™ substrate (Amersham Biosciences Europe, Freiburg, Germany) on a ChemiDoc™ XRS + System (Bio-Rad Laboratories, Munich, Germany).

### Analysis of phenolic compounds

Determination and identification of phenolic compounds were performed according to Roemmelt et al. (2003) with small modifications. Freeze-dried leaves were ground in a ball mill. The extraction was performed by adding 500 µL of methanol containing 3-methoxyflavone as internal standard to 100 mg of powder for a period of 30 min in a cooled ultrasound water bath (7 °C). After centrifugation (10,000*g*, 10 min, 4 °C), the clear supernatant was transferred to an Eppendorf tube. A 10 µL sample of the extract was injected for HPLC analysis. The phenolic compounds were separated on a Nucleosil column (250 × 4 mm, Macherey–Nagel) and eluted with a mixture of H_2_O containing 5 % HCO_2_H (solvent A) and MeOH (solvent B). The following gradient was applied using a flow rate of 0.5 mL/min: 0–5 min, 5 % B; 5–10 min, 5–10 % B; 10–15 min, 10 % B; 15–35 min, 10–15 % B; 35–55 min, 15 % B; 55–70 min, 15–20 % B; 70–80 min, 20 % B; 80–95 min, 20–25 % B; 95–125 min, 25–30 % B; 125–145 min, 30–40 % B; 145–160 min, 40–50 % B; 160–175 min, 50–90 % B; 175–195 min, 90 % B (Treutter et al. 1994). For the quantification of phloridzin the extract was diluted 200-fold with methanol and analyzed using a short column (12.5 × 4 mm I.D.) prepacked with LiChrospher 100 RP18, 5 µm particle size, and a gradient range from 40 to 90 % aqueous methanol. Dihydrochalcones, hydroxycinnamic acids, and flavonols were detected at 280, 320 and 350 nm. The monomeric flavan-3-ols and the procyanidins were estimated using a chemical reaction detection (HPLC-CRD) at 640 nm after postcolumn derivatization with 4-dimethylaminocinnamicaldehyde (DMACA) (Treutter 1989). Quantification was performed as follows: phloridzin and phloretin were available as standard; 3-hydroxyphloridzin was calculated as phloridzin; hydroxycinnamic acids as chlorogenic acid; flavonols as rutin; catechin, epicatechin and procyanidin B2 were available as standards; procyanidin B5 and EB-5 were calculated as procyanidin B2. Peak identification is published elsewhere (Mayr et al. 1995; Roemmelt et al. 2003).

### Identification of 3-hydroxyphloridzin

The putative 3-hydroxyphloridzin peak showed an UV spectrum with *λ*_max_ at 284 nm which is similar with those of phloridzin in front of each is eluted at *t*_R_ = 97 min. The same elution behavior was reported by Leu et al. (2006). The compound was isolated by several analytical HPLC runs and respective fractions were combined. The hydrolysis with glycosidase was performed as described by Regos et al. (2009). MS analysis was performed with a Time-of-Flight mass spectrometer (ToF–MS) (6200 series ToF, Agilent Technologies, Santa Clara, CA, USA). The sample was provided in a 100 µL syringe (Hamilton-Bonaduz, Switzerland), located in a syringe pump (Model 11 Plus, Harvard Apparatus, Hugo Sachs Elektronik, Hugstetten, Germany), which was set to a flow rate of 8 µL/min. Upon injection to the MS electrospray ionization source sample compounds were detected in negative ionization mode. MS conditions were as follows: 80–3200 *m/z* range; 300 °C gas temperature; 3 L/min gas flow; 15 psig nebulizer operating pressure; 100 °C sheath gas temperature; 3 L/min sheath gas flow; 2000 V capillary voltage; 2000 V nozzle voltage; 65 V skimmer voltage and 100 V fragmentor voltage. Besides the determination of the compounds exact molecular weight, fragmentation was induced using the same MS parameters except for an increase of fragmentor voltage to 200 V.

### Determination of the antioxidant activity

The antioxidant activity of methanolic plant extracts was determined according to the 2,2-diphenyl-1-picrylhydrazyl (DPPH) method as described by Brand-Williams et al. (1995) with slight modifications. 0.25 g leaves were grinded with 0.25 g quartz sand and 3 mL methanol for 1.5 min. After centrifuging the homogenate for 10 min at 4 °C and 10,000*g*, the supernatant was transferred in a new reaction tube. 0, 1, 5, 10, 15 and 20 µL methanolic plant extracts were briefly mixed with 2 mL of 0.1 mM methanolic DPPH solution in cuvettes. The absorbance of DPPH was measured after 30 min at 517 nm using methanol as a blank. The values were used for the generation of a linear equation to calculate the amount of methanolic plant extract which leads to a 50 % absorbance decrease. Using a calibration curve with the vitamin E analogon trolox (6-hydroxy-2,5,7,8-tetramethylchroman-2-carboxylic acid) as a standard the antiradical power (ARP) in µmol trolox equivalents (TE)/g fresh weight (FW) was calculated.

### Enzyme activity measurements

Enzyme preparations from apple leaves were obtained and assays were performed as described by Slatnar et al. (2010). In brief, 0.5 g leaves, 0.25 g quartz sand, and 0.25 g Polyclar AT were homogenized with 3 mL 0.1 MTris/HCl (containing 0.4 % Na-ascorbate, pH 7.25) in a pre-cooled mortar. After centrifuging the homogenate for 10 min at 4 °C and 10,000*g*, 400 µL of the supernatant were passed through a gel chromatography column (Sephadex G25 medium, (GE Healthcare, Vienna Austria)). In a final volume of 100 µL: the CHS/CHI assay contained 40 µL crude extract, 5 µL [^14^C]- malonyl-CoA (1.5 nmol), 5 µL [^14^C]-*p*-coumaroyl-CoA (1 nmol) and 50 µL 0.1 M KPi buffer (pH 7.0, 0.4 % Na-ascorbate w/v); the FHT assay contained 0.036 nmol [^14^C]-naringenin, 30 µL crude extract, 5 µL 2-oxoglutarate (1.46 mg/ml H_2_O), 5 µL FeSO_4_·7H_2_O (0.56 mg/ml H_2_O) and 60 µL 0.1 M Tris/HCl (pH 7.25, 0.4 % Na-ascorbate w/v; the FLS assay contained 0.036 nmol [^14^C]-dihydrokaempferol, 30 µL crude extract, 5 mL 2-oxoglutarate (1.46 mg/mL H_2_O), 5 µL FeSO_4_·7H_2_O (0.56 mg/ml H_2_O) and 60 µL buffer 0.1 M Tris/HCl buffer (pH 7.60, 0.4 % Na-ascorbate w/v). In a final volume of 50 µL the DFR assay contained 0.036 nmol [^14^C]-dihydroquercetin, 20 µL crude extract, 5 µL NADPH (4.18 mg/100 mL H_2_O) and 25 mL 0.1 M KPi buffer (pH 6.8, 0.4 % Na-ascorbate w/v). The assays were incubated for 15 min at 30 °C. Protein determination was performed according to Lowry modified by Sandermann and Strominger (1972).

### Fire blight resistance evaluation

Artificial shoot inoculations were performed using a suspension of *E. amylovora*, strain 222 (10^7^ cfu/mL) on own rooted transgenic and non-transgenic control plants grown in the greenhouse as described by Flachowsky et al. (2008). Twenty replicates for each transgenic line were inoculated.

### Scab resistance evaluation

Resistance to *Venturia inaequalis* was tested on greenhouse plants of transgenic lines and non-transgenic control plants grown on their own roots as described by Flachowsky et al. (2010). Fourteen (M803), eighteen (PinS) and twenty (M801, M802) replicates per genotype were used for evaluation. The inoculum consisted of a mixture of scab strains which was prepared from scab-infected leaves. Leaves were harvested from trees of different genotypes, which are known to differ in their reaction to different scab races. These trees were grown in a plot not treated with fungicides in the orchard of the JKI (Dresden, Germany). Scab incidence was scored using the scale of Chevalier et al. (1991).

### Statistical analysis

Quantitative data were subjected to statistical analysis (ANOVA and Duncan’s multiple range test) using the SAS^®^ 9.1 software (SAS institute, Cary, N.C.).

## Results

### Plant transformation and molecular evaluation of transgenic lines

**Fig. 3:**
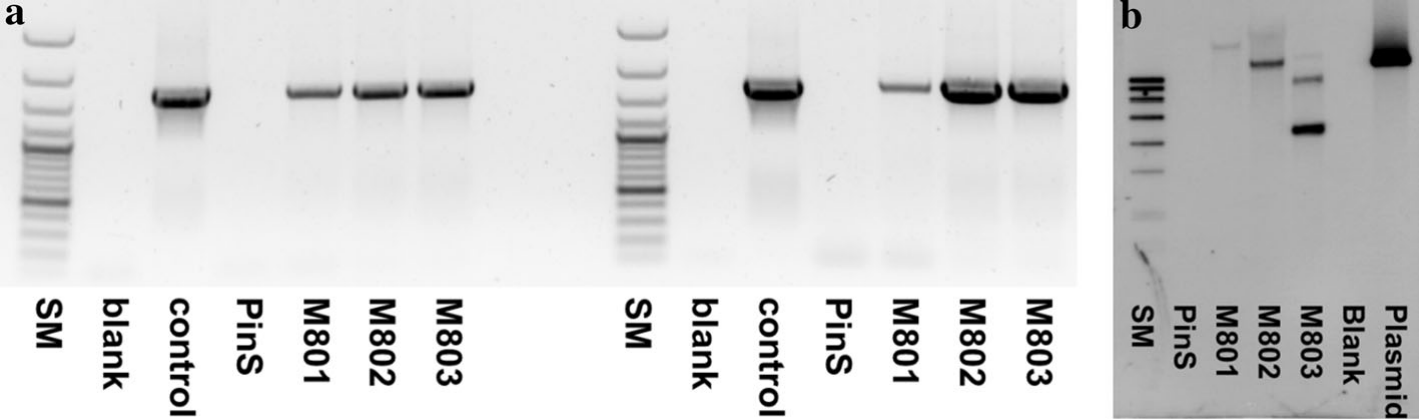
Molecular evaluation of the three transgenic *CsCH3H* apple lines (M801, M802, M803) in comparison to the PinS parent line. **a** PCR-based detection of the *CSCH3H* gene; *left* PCR with genomic DNA, *right* RT-PCR with cDNA from leaves. **b** Detection of integrated T-DNA copies in DNA of the transgenic apple lines by Southern hybridization. *SM* size marker

The ability of CH3H and F3′H to hydroxylate the dihydrochalcone phloretin in position 3 was tested in vitro with recombinant enzymes obtained by heterologous expression in yeast. Both enzymes accepted phloretin as substrate but lower conversion rates were obtained in comparison to the other substrates tested (Suppl. Table S2). Phloretin was a better substrate for CH3H than for F3′H, but conversion rates were still lower than with naringenin despite the high structural similarity between phloretin and isoliquiritigenin (Fig. 2). In order to investigate if CsCH3H can provoke 3-hydroxyphloretin formation *in planta* as well and if these would have an influence on the properties of transgenic apple leaves, a binary plasmid vector producing the CsCH3H enzyme under the control of the CaMV 35S promotor in transformed plant cells was constructed and used for transformation of the *Malus* × *domestica* genotype PinS. Three putative transgenic plants were obtained, which were tested for the presence of transgenic DNA by PCR using primers nptII_F/R for *nptII* and SpeCH3H/CH3HMlu for the *CsCH3H* gene. All tested plants showed fragments of the expected size (Fig. 3a, Suppl. Fig. S1a). The integration of the T-DNA was confirmed by Southern-blot analysis. All plants showed hybridization signals for *CsCH3H* (Fig. 3 b). Signals with specific probes for *nptII*, however, were only obtained with two of the three lines (Suppl. Fig. S1b). The plants were vegetatively propagated to establish three transgenic lines (M801–M803).

### Polyphenol analysis

**Fig. 4:**
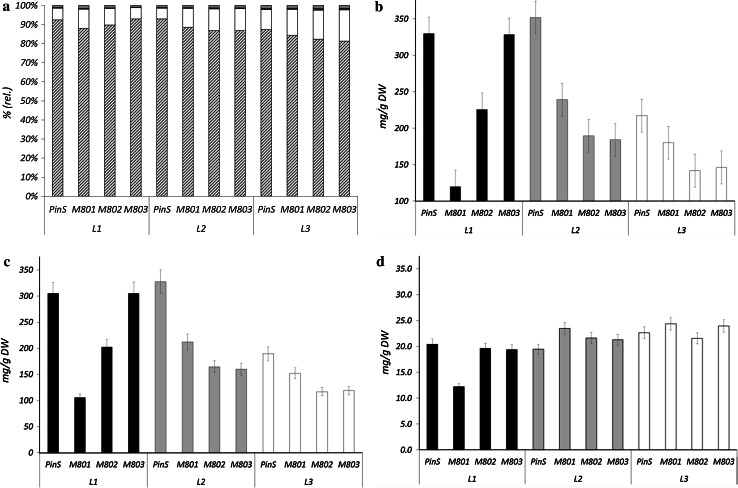
Polyphenol (*hatched* dihydrochalcones, *white* flavonols, *grey* flavanols, *black*: hydroxyl cinnamic acids) distribution (**a**) and concentrations of polyphenols (**b**), dihydrochalcones (**c**), and flavonols (**d**) in the first (L1, *black*), second (L2, *grey*) and third (L3, *white*) leaves in three transgenic *CsCH3H* apple lines (M801, M802, M803) in comparison to the PinS parent line

**Fig. 5:**
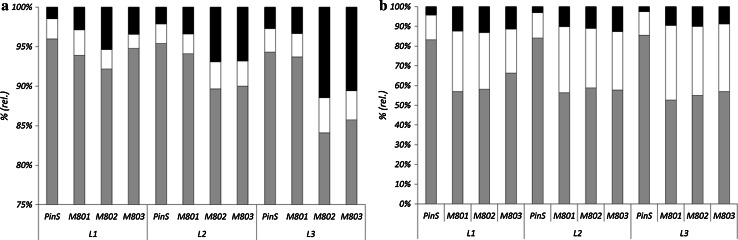
Relative concentrations of dihydrochalcones (**a** *grey* phloridzin, white: phloretin, *black* 3-hydroxyphloridzin) and flavan 3-ols (**b** *grey* dimeric procyanidins B2, B5 and trimeric procyanidin EB-5, *white* epicatechin, *black* catechin) in the first (L1), second (L2) and third (L3) leaves of three transgenic lines M801, M802, M803 in comparison to the parent line PinS

In comparison to the parent line PinS, the transgenic lines showed a decreased content of soluble polyphenols (Fig. 4b). Whereas PinS accumulated between 217 and 350 mg soluble polyphenols/g DW in the leaves, only 120–328 mg were found in the transgenic lines (Suppl. Table S3). The transgenic lines accumulated lower concentrations of dihydrochalcones (Fig. 4c), hydroxycinnamic acids and soluble flavan 3-ols in comparison to the control (Suppl. Table S3). The flavonol content, in contrast, remained almost unchanged (Fig. 4d). Despite the generally lower polyphenol contents in the transgenic lines, the distribution of the polyphenol classes was quite similar in parent and transgenic lines (Fig. 4a). Dihydrochalcones were the predominant polyphenol class with 80–90 % of the soluble polyphenol content followed by flavonols (approx. 6–10 %), low amounts of hydroxycinnamic acids and soluble flavan 3-ols (both below 2 %) (Fig. 4a). The prevalent dihydrochalcone in all lines was phloridzin, whereas 3-hydroxyphloridzin remained below 2 % of the total dihydrochalcones in the parent line. In contrast, all transgenic lines showed an increased 3-hydroxyphloridzin content, which was particularly distinct in lines M802 and M803 where an increase of up to 11.5 % of the total dihydrochalcones was observed (Fig. 5). The relative content of 3-hydroxyphloridzin increased with leaf age in all lines (Fig. 5, Suppl. Table S3). Highest amounts of 3-hydroxyphloridzin in terms of absolute values and relative contents were found in line M802, whereas line M801 showed the lowest increase in 3-hydroxyphloridzin accumulation. The presence and increased accumulation of 3-hydroxyphloridzin was unequivocally confirmed by HPLC and LC–MS with a commercially available reference compound for 3-hydroxyphloretin, which in turn could not be detected in any of the samples. The isolated compound gave the molecular mass of *m/z* [M-H]^−^ 451.1239, which is highly accurate to the calculated *m*/*z* of phloridzin (Suppl. Table S4). Furthermore fragmentation experiments demonstrated the cleavage of a hexose moiety (~162 Da), thereby generating a fragment ion with *m/z* 289.0725, which can be ascribed to the presence of 3-hydroxyphloretin (Suppl. Fig. S2, Suppl. Table S4). In addition to the dihydrochalcones, the soluble flavan 3-ols composition was altered in all transgenic lines in comparison to the parent line PinS. The relative contents of the monomeric flavan 3-ols catechin and epicatechin were increased in comparison to the flavan 3-ol dimers proanthocyanidin B2 and proanthocyanidin B5 and the flavan 3-ol trimers proanthocyanidin E-B5 (Fig. 5, right). Antioxidant capacity measured in the DPPH assay was almost comparable but a slight increase with leaf age was observed in all transgenic lines (Suppl. Fig. S3).

**Fig. 6:**
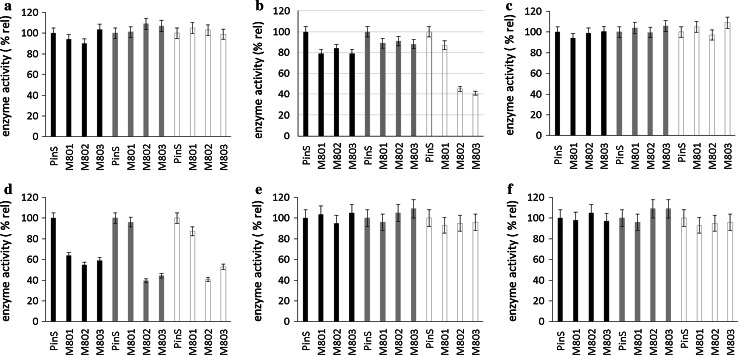
Relative enzyme activities of transgenic *CsCH3H* apple lines (M801, M802, M803) in comparison to the parent line PinS in the first (L1, *black*), second (L2, *grey*) and third (L3, *white*) leaves. Enzyme activities were calculated in relation to the values determined for the parent line in the respective leaf stage. **a** Phenyl alanine ammonia lyase [100 % correspond to 0.8 (L1), 0.5 (L2), and 0.4 (L3) µmol^−1^ kg^−1^]. **b** Chalcone synthase/chalcone isomerase [100 % correspond to 3.6 (L1), 2.0 (L2), and 1.8 (L3) µmol^−1^ kg^−1^]. **c** Flavanone 3-hydroxylase [100 % correspond to 1.5 (L1), 1.5 (L2), and 1.0 (L3) µmol^−1^ kg^−1^]. **d** Dihydroflavonol 4-reductase) [100 % correspond to 1.9 (L1), 1.6 (L2), and 2.0 (L3) µmol^−1^ kg^−1^]. **e** Flavonol synthase (100 % correspond to 0.2 µmol^−1^ kg^−1^ for all leave stages). **f** Phloretin 2′-*O*-glucosyl transferase (100 % correspond to 1.0 (L1), 1.3 (L2), and 2.0 (L3) µmol^−1^ kg^−1^)

**Fig. 7:**
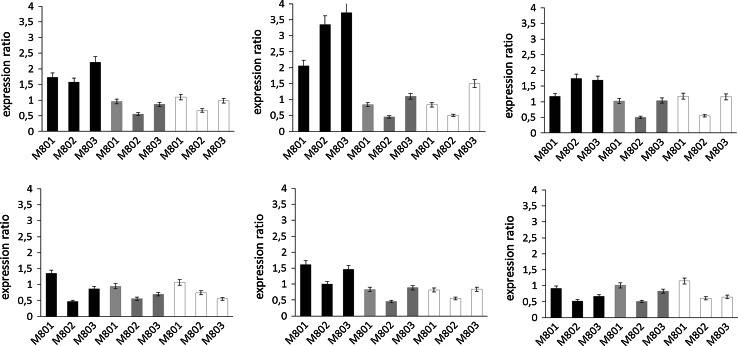
Expression of anthocyanidin synthase (*left*), anthocyanidin reductase (*centre*) and flavonoid 3-*O*-glucosyltransferase (*right*) in transgenic *CsCH3H* apple trees (M801, M802, M803) in comparison to the parent line PinS (expression ratio 1) in the first (L1, *black*), second (L2, *grey*) and third (L3, *white*) leaves. Expression was measured in comparison to the housekeeping genes *elongation factor* (*above*) and *glycerinaldehyde 3*-*phosphate dehydrogenase* (*below*)

Apart from polyphenol concentrations we measured the activities of the key enzymes of the flavonoid pathway including phenylalanine ammonium lyase (PAL), chalcone synthase/chalcone isomerase (CHS/CHI), flavanone 3-hydroxylase (FHT), dihydroflavonol 4-reductase (DFR), flavonol synthase (FLS) and phloretin 2′-*O*-glucosyl transferase (P2′GT). CH3H was not included because the use of frozen leaves impeded the detection of the activity of membrane bound enzymes. In comparison to the parent line, the activities of PAL, FHT, FLS and P2′GT remained almost unchanged in all transgenic lines (Fig. 6). DFR activity clearly decreased in all transgenic lines in comparison to the parent line, whereas CHS/CHI showed a distinct decrease only in the third leaves, whereas in the first and second leaves the decrease was only modest (Fig. 6). To shed light on the flavan 3-ol formation we measured the gene expression of *anthocyanidin synthase* (*ANS*), *anthocyanidin reductase* (*ANS*) and *flavonoid 3*-O-*glucosyl transferase (F3GT)* by qPCR. No strong modulation of any of these genes in the transgenic lines was observed in comparison with the parent line PinS (Fig. 7). This was confirmed by normalization against two housekeeping genes, *elongation factor 1 alpha* and *glycerinaldehyde 3*-*phosphate dehydrogenase* (Fig. 7). The expression ratio in comparison to PinS remained between 0.5 and 2 for all genes with exception of *ANR*, which showed slightly higher expression ratios in the first leaves when normalization was done with *elongation factor 1 alpha* as housekeeping gene. The differences obtained with the two housekeeping genes can be explained by slight expression differences in the two housekeeping genes (Suppl. Fig. S4).

### Evaluation of the disease resistance of the transgenic *CsCH3H* lines

**Fig. 8:**
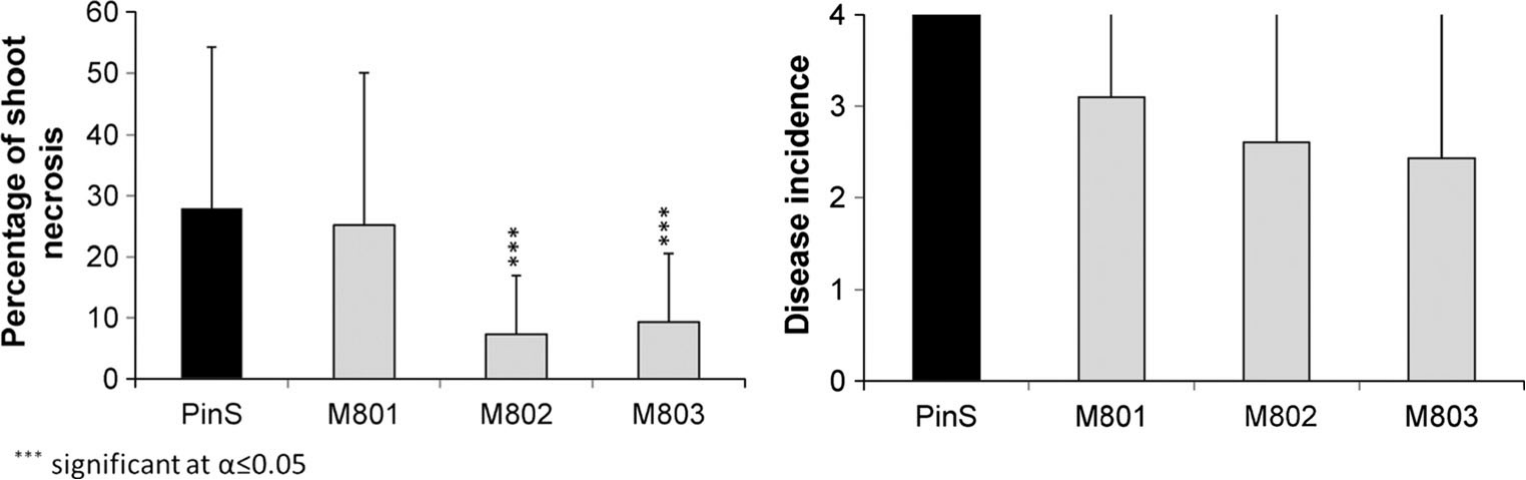
Evaluation of the disease susceptability of the transgenic *CsCH3H* lines (M801, M802, M803) in comparison to the parent line PinS. *Left* Percentage of shoot necrosis after inocculation with the *Erwinia amylovora* strain Ea222. *Right* Disease incidence according to Chevalier et al. (1991) after infection of the leaves with a mixture of *Venturia inaequalis* strains present in the open field

The transgenic *CsCH3H* lines and the parental genpotype PinS were evaluated for their resistance to fire blight and apple scab. 20 shoots each were inoculated with *E. amylovora* strain 222. Six weeks after inoculation plants were evaluated on disease symptoms. Inoculated shoots of the transgenic lines showed reduced mean percentage of shoot necrosis as expected in the case of reduced susceptibility to fire blight (Fig. 8, left). Statistically significant differences between transgenic plants and non-transgenic wild type plants at *α* ≤ 0.05 were found for lines M802 and M803. No statistically significant differences were found for the mean length of the shoot re-growth suggesting that all plants were in a physiologically comparable state.

For the evaluation of resistance to scab up to twenty replicates of the transgenic *CsCH3H* lines and the non-transgenic control were inoculated with *Venturiainaequalis* as described. On average, three leaves per plant of the non-transgenic genotype PinS were scored with 4 (sporulation) and all replicates showed susceptible reaction. The number of leaves scored with 4 was lower in transgenic lines M801, M802 and M803 with one leaf per plant on average (Fig. 8, right). Nevertheless, six, six and seven replicates of M801, M802 and M803 showed no compatible interaction. The mean incidence was obviously lower in transgenic plant, but statistically significant differences could not been found. Regarding that all transgenic lines showed susceptible interaction with scab but less severity the development of the disease on transgenic plants seemed to be slowlier but resistance was not introduced.

## Discussion

Dihydrochalcones are accumulated in *Malus* sp. (apple) in such large amounts that it appears unlikely that this should be just a result of polyphenolic waste storage in vacuoles. Knowledge of their biosynthesis is an essential precondition for understanding the physiological role of dihydrochalcones because this allows studying activities of corresponding enzymes and genes as a response to biotic and abiotic stresses. Despite the large amounts of dihydrochalcones present in *Malus* sp. their formation has been studied only recently. There still remain a lot of open questions such as the identity of the dehydrogenase providing the precursor for the formation of the dihydrochalcone structure (Gosch et al. 2009; Dare et al. 2013; Ibdah et al. 2014) and of the enzymes involved in 3-hydroxyphloretin formation. In the present study we created transgenic apple lines overexpressing the CH3H from the ornamental plant *Cosmos sulphureus*, which accepts phloretin as substrate in vitro. This allowed us to study the ability of CH3H to hydroxylate dihydrochalcones *in planta*. In addition, this shed new light on the 3-hydroxyphloridzin biosynthesis in apple and allowed to evaluate the physiological relevance of increased 3-hydroxyphloridzin levels for pathogen defence.

The three transgenic lines accumulated higher amounts of 3-hydroxyphloridzin than the parent line, thereby confirming that CH3H accepts dihydrochalcones as substrates not only in vitro but also *in planta*. However, a maximal increase of up to 11.5 % of the dihydrochalcone content was observed. This confirmed that dihydrochalcones are not as good substrates for CH3H as chalcones. Only 3-hydroxyphloridzin, but no 3-hydroxyphloretin accumulation was observed, indicating that the highly reactive 3-hydroxyphloretin is immediately converted to hydroxyphloridzin to avoid undesired cell damage and that the glucosyl transferases of apple accept 3-hydroxyphloretin besides phloretin as substrates. The parent line PinS accumulated between 4.5 and 7 mg/g DW 3-hydroxyphloridzin in the leaves. Considering the lower conversion rates of F3′H compared to CH3H observed in vitro with phloretin as substrate, it seems to be possible that F3′H is involved in the biosynthesis of the constitutive 3-hydroxyphloridzin levels in apple.

All transgenic *CsCH3H* lines generally showed lower concentrations of polyphenols. Flavonol concentrations were less affected which could be explained by the lower DFR activities but almost unchanged FLS activities in transgenic lines compared to the parent line. Because DFR and FLS compete for dihydroflavonols as common substrates it seems likely that a bottleneck at the stage of DFR increases the availability of precursors for flavonol formation and decreases the concentrations of downstream products such as flavan 3-ols. The decrease of dihydrochalcone concentrations could be partially explained by the lower CHS/CHI activities observed. However, as this was a rather decent decline in comparison to DFR activity we assume that other, unknown factors must be responsible.

Apple polyphenols were shown to possess strong antioxidant and radical scavenging activities (Lu and Foo 2000). Highest activities were observed with flavonols and flavan 3-ols, and 3-hydroxyphloridzin showed considerably higher effects than phloridzin (de Bernonville et al. 2010). No striking differences in the antioxidant capacity were observed between the transgenic lines and the parent lines. As the flavonol concentrations remained unchanged and the total polyphenol concentration decreased drastically, it seems likely that the increased 3-hydroxyphloridzin concentrations in the transgenic lines compensated the decrease resulting from the lower polyphenol concentrations. This assumption is supported by the fact that both 3-hydroxyphloridzin concentrations and the antioxidant capacity increased with leaf age.

The transgenic lines showed reduced susceptibility to fire blight and apple scab, which are the main bacterial and fungal diseases of apple. Two of the three lines showed a statistically significant reduced percentage of shoot necrosis after infection with *E. amylovora*. All lines showed a lower disease severity after infection with *V. inaequalis*. The most striking changes in the transgenic lines with respect to polyphenol composition, was the increase of 3-hydroxyphloridzin, the higher amounts of monomeric flavan-3-ols at the expense of oligomeric flavan 3-ols and the generally lower polyphenol concentrations. As polyphenols are assumed to have significant roles in disease resistance (Treutter 2006) the reduced concentrations are probably not responsible for the observed effects. The flavan 3-ols could be of relevance however all three lines showed a similar reduction in flavan 3-ols but different levels of susceptibility. Interestingly the 3-hydroxyphloridzin concentrations correlate very well with the observed decrease in disease susceptibility. Both, lines M802 and M803 showed higher 3-hydroxyphloridzin concentrations in the first, the second as well as in the third leaves and a significant lower percentage lesion length after inoculation with fire blight than the parental line PinS, whereas line M801 showed similar 3-hydroxyphloridzin concentrations as parental line PinS and only a slight but not significant reduction of shoot necrosis. Additionally, the mean incidence of scab on the leaves was lower for transgenic lines M802 and M803 than for M801 and PinS.

The transgenic lines harbouring *C. sulphureus* CH3H have been created as model plants which allowed for the first time to study the physiological relevance of 3-hydroxyphloridzin. The constitutive overexpression of the *C. sulphureus* CH3H in transgenic apple plants resulted in an increase of 3-hydroxyphloridzin formation *in planta* which seems to be correlated with reduced susceptibility or a slowlier development of disease symptoms to two economically important apple diseases. However, the transgenic lines unexpectedly showed lower polyphenol concentrations than the parent lines. The effect of such a drastic change in the polyphenol composition on the apple fruit (e.g. quality, taste, shelf life) needs to be tested in the future. In addition our study has demonstrated that 3-hydroxyphloretin can be created by cytochrome P450 dependent monooxygenases from the flavonoid pathway and has enlarged the substrate spectrum known to be accepted by F3′H. If the MalusF3′Hs are involved in the 3-hydroxyphloridzin biosynthesis in apple will be investigated in a future study.

### *Author contribution statement*

OSH, HF, IR, SM, CK, SF, MZA, CG, AP, KR, MVH, DT performed research, OSH, HF, KS and HH wrote the manuscript, HH designed the study.

## Electronic supplementary material

Below is the link to the electronic supplementary material.

Supplementary material 1 (DOCX 385 kb)

## Abbreviations

CH3H: Chalcone 3-hydroxylase
F3′H: Flavonoid 3′-hydroxylase
PPO: Polyphenol oxidase
