# Supplementary material for: Transgenic apple plants overexpressing the chalcone 3-hydroxylase gene of Cosmos sulphureus show increased levels of 3-hydroxyphloridzin and reduced susceptibility to apple scab and fire blight
Source: Planta. 2016 Feb 19;243:1213–24. doi: 10.1007/s00425-016-2475-9 (PMC4837221; doi:10.1007/s00425-016-2475-9)
Supplement: Supplementary file 1 — Supplementary material 1 (DOCX 385 kb) [file 425_2016_2475_MOESM1_ESM.docx]

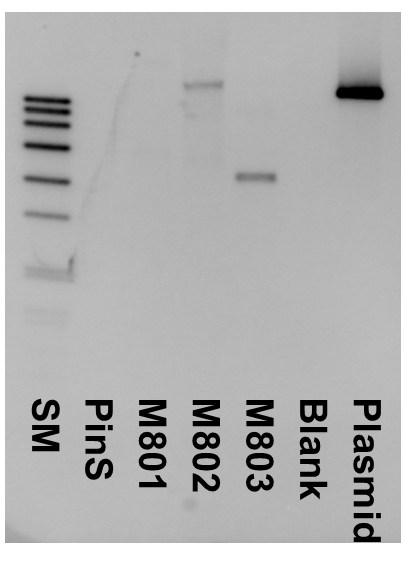

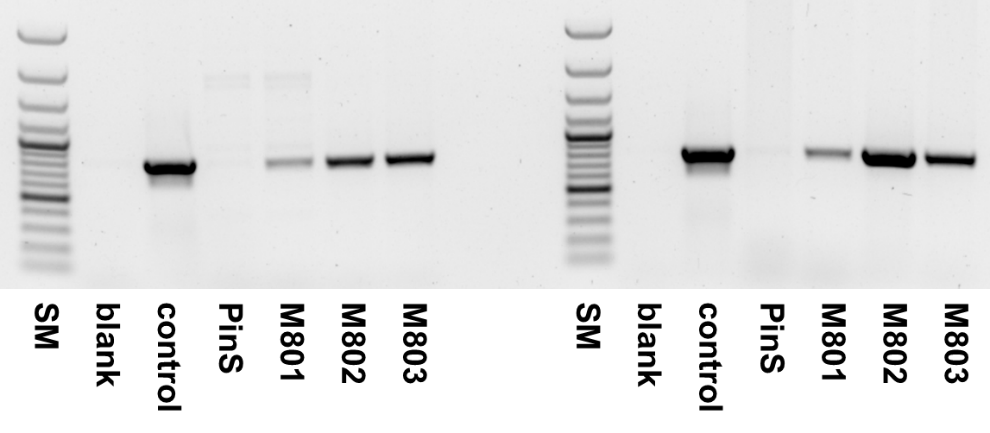


**Supplementary Figure S1** Molecular evaluation of the three transgenic *CsCH3H* apple lines in comparison to the PinS control. a: PCR-based detection of the *nptII* selection; left PCR with genomic DNA, right RT-PCR with cDNA from leaves. b: Detection of integrated T-DNA copies in DNA of the *CsCH3H* transgenic apple lines by Southern hybridization. abbrev: SM: Size marker

289.0718

451.1246

**Supplementary Figure S2** Mass spectra of 3-hydroxyphloridzin showing (A) the accurate mass of the compound and (B) the loss of a hexose and 3-hydroxyphloretin pseudomolecular ion


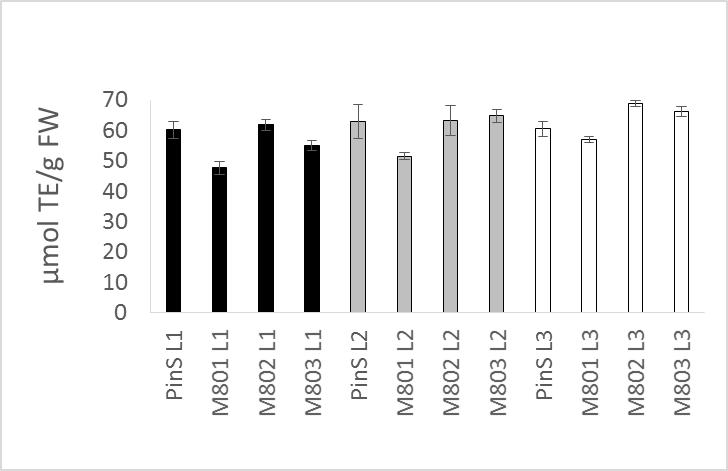


**Supplementary Figure S3** Antiradical power (µmol TE/g FW) determined by the DPPH method in extracts of the first (L1, black), second (L2, grey) and third (L3, white) leaves of three transgenic *CsCH3H* apple lines (M801, M802, M803) in comparison to the PinS parent line

**Supplementary Figure S4** Expression of *elongation factor* normalized against the housekeeping gene *glycerinaldehyde 3-phosphate dehydrogenase* in transgenic *CsCH3H* apple trees in comparison to the parent line PinS (expression ratio 1) in the first (L1, black), second (L2, grey) and third (L3, white) leaves

**Supplementary Table S1** List of primers used

| **Primer ID** | **Sequence (5´>3´direction)** | **T_m_ (°C)** | **Size (bp)** | **Source  (Acc. no./pub.)** |
| --- | --- | --- | --- | --- |
| *Mal.EF.F* | TACTGGAACATCACAGGCTGAC | 60.3 | 308 | Vanblaere et al. (2011) |
| *Mal.EF.R* | TGGACCTCTCATCATGTTGT | 55.3 |  |  |
| *Mal.ANS.F* | GTCATGCACATTGGGGATACACTT | 61.0 | 102 | AF117269 |
| *Mal.ANS.R* | CCATGAAATCCTCACCTTTTCCTTG | 61.3 |  |  |
| *Mal.ANR.F* | TTTTGGCTGAGAAAGAATCTGCATC | 59.7 | 100 | DQ099803 |
| *Mal.ANR.R* | TGGGGGTATCTTTTGTTGAGGAACT | 61.3 |  |  |
| *Mal.FGT.F* | TGTACATAAGCTTCGGGACAGTG | 60.6 | 102 | DQ156906 |
| *Mal.FGT..R* | TGATAGACCACAAGAAGGGTGCT | 60.6 |  |  |
| *Mal.GAPDH.F* | CACCACCGACTACATGACCTA | 62.5 | 99 | MDC007381 |
| *Mal.GAPDH.R* | AAGAGAAGGGTCTTGGAGTCCT | 63.3 |  |  |
| *SpeCH3H^*^* | CCCTC**ACTAGT**CCCAACAACTCTTACTA | 65.1 | 1639 | p9u10-35S::CH3H |
| *CH3HMlu^*^* | CCTT**ACGCGT**TGAAACAGGAACTCAG | 64.8 |  |  |
| *nptII_F* | ACAAGATGGATTGCACGCAGG | 59.8 | 780 | Flachowsky et al. 2007 |
| *nptII_R* | AACTCGTCAAGAAGGCGATAG | 57.9 |  |  |

* restriction sites for *Spe*I and *Mlu*I which were used for cloning the CH3H cDNA into p9u10-35S are written in bold and underlined

**Supplementary Table S2** Conversion rates of recombinant CH3H and F3'H using the 6'-deoxychalcone isoliquiritigenin, the flavanone naringenin and the dihydrochalcone phloretin as substrates

|  | **% Conversion rate with** | | |
| --- | --- | --- | --- |
| **Recombinant enzyme** | **isoliquiritigenin** | **naringenin** | **phloretin** |
| CH3H (NCBI FJ216429) | 92.5±2 | 40.6±4 | 30.2±2 |
| F3'H (NCBI FJ216426) | - | 45.6±3 | 10.4±3 |

**Supplementary Table S4** m/z contained in fractionated sample detected with a ToF-MS in negative ionization mode. Deviation to the calculated molecular weight prior (100 V fragmentor voltage) and after fragmentation (200 V) is given in ppm

|  | **Detected m/z** | **Calculated m/z** | **ppm** |
| --- | --- | --- | --- |
| [Phloridzin-H]^-^ | 451.1239 | 451.1246 (C_21_H_24_O_11_) | 1.55 |
| [3-hydroxyphloretin-H]^-^  (200 V fragmentor voltage) | 289.0725 | 289.0718 (C_15_H_14_O_5_) | -2.42 |

**Supplementary Table S3** Overview of the polyphenol composition in the first (L1), second (L2) and third (L3) leaves
in three transgenic *CsCH3H* apple lines (M801, M802, M803) in comparison to the PinS parent line.

| **mg/g DW** | | | | | | | | | | | | | | |
| --- | --- | --- | --- | --- | --- | --- | --- | --- | --- | --- | --- | --- | --- | --- |
| **Sample** | | **Total polyphenols** | **Total dihydro-**  **chalcones** | **phloridzin** | **phloretin** | **3-Hydroxy- phloridzin** | **Total flavonols** | **Hydroxycinnamic acids** | **Flavan 3-ols** | **Epicatechin** | **Catechin** | **Procyanidin B2** | **Procyanidin B5** | **Procyanidin E-B5** |
| Leaf 1 | PinS | 329 ± 16 | 305 ± 15 | 293 ± 15 | 1.5 ±0.2 | 4.5±0.5 | 20.4 ± 1 | 3.3 ± 0.3 | 1.2 ± 0.2 | 0.18 ± 0.02 | 0.06 ± 0.01 | 0.04 ± 0.01 | 0.8 ± 0.04 | 0.31 ± 0.02 |
|  | M801 | 120 ± 6 | 105 ± 5 | 99 ± 5 | 0.6 ±0.1 | 3.0 ±0.2 | 12.2 ± 0.6 | 1.9 ± 0.2 | 0.2 ± 0.05 | 0.12 ± 0.01 | 0.05 ± 0.01 | 0.02 ± 0.00 | 0.15 ± 0.01 | 0.05 ± 0.01 |
|  | M802 | 226 ± 11 | 203 ± 10 | 187 ± 9 | 0.8±0.1 | 10.9±1 | 19.6 ± 1 | 2.9 ± 0.3 | 0.4 ± 0.05 | 0.18 ± 0.01 | 0.08 ± 0.02 | 0.03 ± 0.01 | 0.28 ± 0.01 | 0.05 ± 0.01 |
|  | M803 | 329 ± 16 | 305 ± 15 | 289 ± 14 | 1.1±0.2 | 10.4 ±1 | 19.4 ± 1 | 2.8 ± 0.3 | 0.7 ± 0.05 | 0.24 ± 0.03 | 0.12 ± 0.02 | 0.06 ± 0.01 | 0.51 ± 0.01 | 0.15 ± 0.01 |
| Leaf 2 | PinS | 352 ± 18 | 327 ± 16 | 312±16 | 2.1±0.2 | 7.0 ±0.7 | 19.5 ± 1 | 3.5 ± 0.3 | 1.3 ± 0.05 | 0.21 ± 0.03 | 0.05 ± 0.01 | 0.05 ± 0.01 | 0.98 ± 0.02 | 0.30 ± 0.01 |
|  | M801 | 239 ± 12 | 212 ± 11 | 199 ± 10 | 1.6±0.2 | 7.2±0.8 | 23.5 ± 1 | 2.7 ± 0.3 | 0.6 ± 0.05 | 0.35 ± 0.03 | 0.11 ± 0.02 | 0.07 ± 0.02 | 0.42 ± 0.01 | 0.11 ± 0.01 |
|  | M802 | 190 ± 10 | 165 ± 8 | 148 ± 7 | 1.8±0.2 | 11.4 ±1 | 21.6 ± 1 | 2.4 ± 0.3 | 0.5 ± 0.05 | 0.29 ± 0.03 | 0.10 ± 0.02 | 0.07 ± 0.02 | 0.40 ± 0.01 | 0.09 ± 0.01 |
|  | M803 | 184 ± 9 | 160 ± 8 | 144 ± 7 | 1.2±0.1 | 10.9 ±1 | 21.3 ± 1 | 2.4 ± 0.3 | 0.4 ± 0.05 | 0.21 ± 0.03 | 0.09 ± 0.01 | 0.04 ± 0.01 | 0.29 ± 0.01 | 0.07 ± 0.00 |
| Leaf 3 | PinS | 217 ± 11 | 190 ± 10 | 179 ±9 | 0.6±0.1 | 5.1±0.5 | 22.7 ± 1 | 3.5 ± 0.4 | 1.2 ± 0.1 | 0.16 ± 0.01 | 0.03 ± 0.01 | 0.06 ± 0.01 | 0.89 ± 0.02 | 0.22 ± 0.01 |
|  | M801 | 180 ± 9 | 152 ± 8 | 143 ± 7 | 0.9±0.1 | 5.1±0.5 | 24.4 ± 1 | 2.6 ± 0.3 | 0.6 ± 0.05 | 0.43 ± 0.04 | 0.11 ± 0.02 | 0.11 ± 0.02 | 0.36 ± 0.01 | 0.12 ± 0.01 |
|  | M802 | 142 ± 7 | 117 ± 6 | 98 ± 5 | 1.8±0.2 | 13.4±1 | 21.6 ± 1 | 2.4 ± 0.3 | 0.6 ± 0.05 | 0.36 ± 0.04 | 0.10 ± 0.03 | 0.11 ± 0.02 | 0.37 ± 0.01 | 0.08 ± 0.01 |
|  | M803 | 147 ± 7 | 119 ± 6 | 102 ± 5 | 1.1±0.2 | 12.6±1 | 24.0 ± 1 | 2.4 ± 0.3 | 0.6 ± 0.05 | 0.36 ± 0.04 | 0.09 ± 0.03 | 0.11 ± 0.02 | 0.36 ± 0.01 | 0.13 ± 0.02 |
